# Explore antibody repertoire in the era of AI: Antibody repertoire analysis empowered by machine learning

**DOI:** 10.3724/abbs.2025230

**Published:** 2025-12-16

**Authors:** Yudi Zhang, Hefei Wang, Chencheng Liu, Fei-Long Meng

**Affiliations:** 1 Key Laboratory of Systems Health Science of Zhejiang Province School of Life Science Hangzhou Institute for Advanced Study University of Chinese Academy of Sciences Hangzhou 310024 China; 2 Key Laboratory of RNA Innovation Science and Engineering Shanghai Academy of Natural Sciences (SANS) Shanghai Institute of Biochemistry and Cell Biology Center for Excellence in Molecular Cell Science Chinese Academy of Sciences University of Chinese Academy of Sciences Shanghai 200031 China

**Keywords:** antibody repertoire, antibody clonotype, deep learning, vaccine development, immunologic diagnosis

## Abstract

The diverse antibodies of adaptive immunity comprise an antibody repertoire that combats various pathogens. This repertoire is shaped by both intrinsic antibody gene diversification and extrinsic cellular selection. Conversely, an antibody repertoire contains multiple layers of immunological information, including the history of pathogen exposure. High-throughput sequencing-based antibody repertoire cloning approaches have revealed unexpected features of adaptive immunity. However, our understanding of antibody repertoire data is still in its infancy. In this review, we introduce the emerging concepts and discuss the application of deep learning approaches to understanding antibody repertoires. First, we introduce the definition and functional features of antibody clonotype. Next, we review the evolution of antibody clonotypes and discuss potential antibody repertoire-directed vaccination approaches. Lastly, we summarize the application of deep learning in predicting antibody binding, generating specific antibodies, and making immunologic diagnoses. Recently, artificial intelligence (AI) has made revolutionary progress in biology. Leveraging high-dimensional antibody repertoire information, deep learning models have the potential to transform our understanding of antibody repertoire.

## Introduction

**Figure 1:**
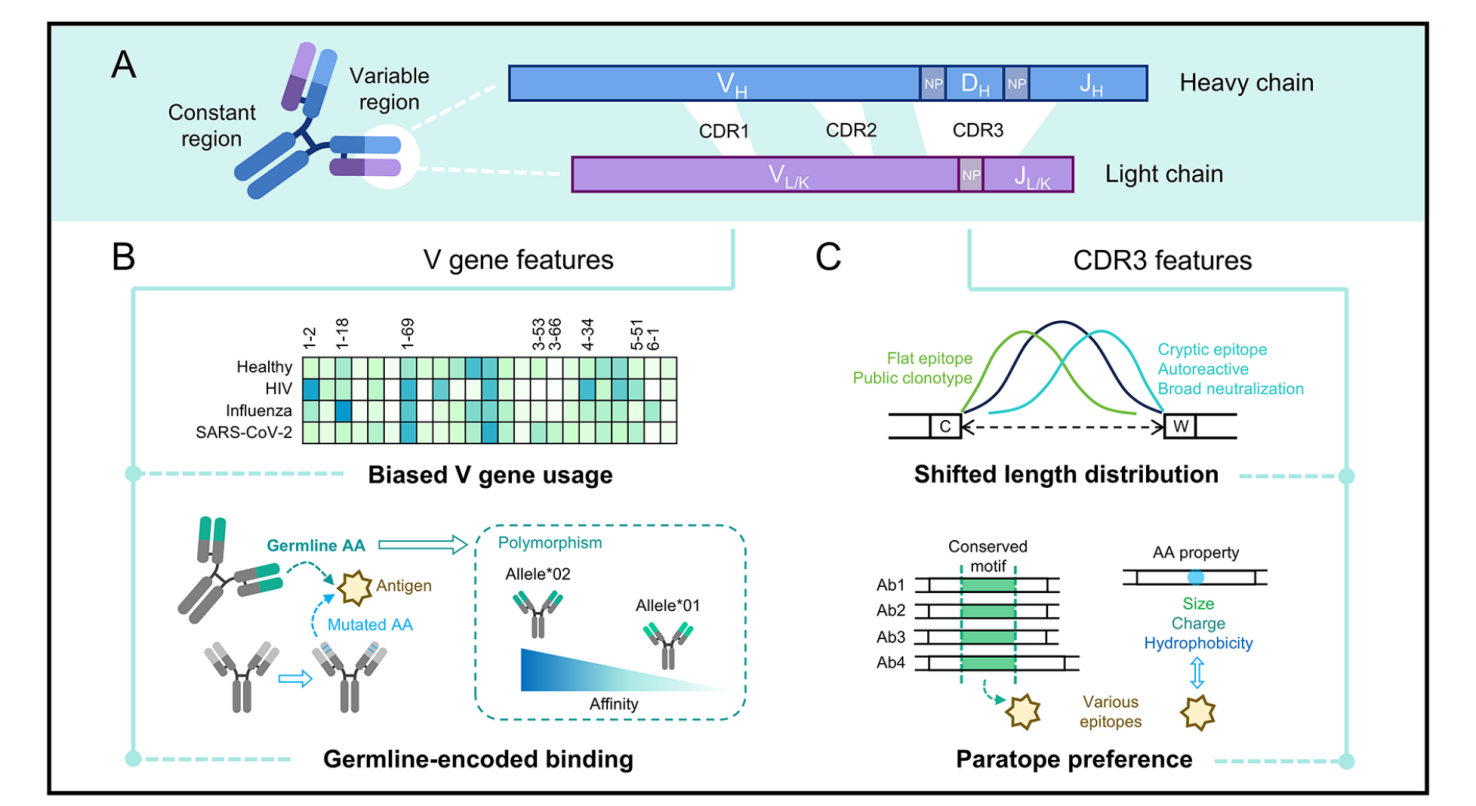
Figure 1 Features of functional antibodies (A) Human antibodies consist of two heavy and two light chains, each comprising variable and constant regions. The variable region is generated by V(D)J recombination and contains three hypervariable CDRs. During recombination, non-template/palindromic (NP) nucleotides are inserted between V-D and D-J junctions in heavy chains, and between V-J junctions in light chains. Functional antibodies display distinct features in their V gene segments and CDR3s. (B) Antigen-specific antibodies often exhibit biased V gene usage compared with the overall repertoire in healthy individuals. Germline-encoded binding motifs and allelic variants can modulate antibody affinity. (C) CDR3 length distributions differ among antibodies with distinct functions. Conserved paratope motifs and enrichment of amino acids with specific physicochemical properties are observed within CDR3 regions.

Antibodies are essential components of the immune system that recognize a wide array of pathogens due to their extraordinary diversity in **humoral immunity**. They function through antigen recognition by their variable domains and through effector functions by their constant domains [ 1– 3] ( Figure 1A). The pool of antibodies expressed by an individual is called **antibody repertoire**, which provides full protection from environmental pathogens. Since the 1980s, antibody research has progressed from screening for specific monoclonal antibodies to exploring the functionality of antibody repertoires [ 4– 7]. In the era of artificial intelligence (AI), the field has advanced to leveraging machine learning (ML) to analyze antibody repertoires in physiological and pathological settings [ 8– 10]. Thus, the rich information embedded in antibody repertoires is beginning to emerge.

The development of intrinsic antibody diversity involves two steps: an antigen-independent step during the early stage of B cell development and an antigen-dependent step during the humoral immune response [ 3, 11, 12]. In the former primary diversification step, the variable regions of the heavy and light chains are assembled through **V(D)J recombination** [13] ( Figure 1A). This process involves joining germline gene segments, inserting and/or deleting nucleotides in junctional regions, and pairing the heavy and light chains, which ultimately enables naïve B cells to express up to 10 ^15^ distinct antibodies [ 13, 14]. The assembled variable region is structurally organized into four framework regions (FWRs) and three hypervariable complementarity-determining regions (CDRs). The flexible, loop-structured CDRs primarily participate in antigen binding. Of these, CDR3 spans the junctional region and exhibits the greatest degree of diversity. Some of these naïve antibodies possess an inherent capacity for antigen recognition. In the latter secondary diversification step, antibody genes are further diversified in the antigen-activated mature B cells. With the help from other immune cells, B cells can form **germinal center (GC)** structures upon antigen stimulation and enhance affinity for their target antigens through the accumulation of **somatic hypermutation (SHM)** in the variable regions [ 11, 15– 17]. In addition, **class switching recombination (CSR)** further increases the degree of antibody diversity by altering the constant region, conferring different effector functions to the same antibody variable region [ 17– 19]. In this antigen-dependent step, B cell clonal lineages that recognize various pathogens are generated and affinity-matured B cells are developed.

Extrinsic cellular selection of B cells further shapes the antibody repertoire by selecting against autoantigens and for non-self antigens. Due to immune memory, almost all past or ongoing diseases theoretically leave unique imprints on the adaptive immune system [ 20, 21]. Indeed, compelling evidence suggests that antibodies recognizing certain pathogens can be found in the antibody repertoires of patients and healthy individuals alike [ 21– 24]. These observations underscore the considerable potential of antibodies as valuable molecular biomarkers for disease diagnosis [25]. Furthermore, baseline features that predict vaccine efficacy have been proposed [26], and evaluating vaccine efficacy necessitates greater precise features beyond antibody titer [ 27, 28].

Using antibody discovery technologies, researchers have isolated a large number of antibodies targeting specific antigens from patients and experimental animal models. Through sequence and structure analyses, they have elucidated function-related antibody features, including germline gene usage, CDR3 sequences, and SHM frequency. These features reveal the trajectory of antibody affinity maturation and guide workflows for functional antibody discovery and vaccine design [29]. In the 2010s, high-throughput B cell receptor (BCR) repertoire sequencing became popular, unlocking vast amounts of antibody sequence information [ 30– 37]. It enables the computational prediction of promising binders, significantly reducing experimental costs [ 32, 38, 39]. However, extracting biomarkers from the immense complexity of antibody repertoire data requires sophisticated, computationally intensive analytical approaches. In this regard, AI, which has demonstrated exceptional performance in protein structure prediction and function optimization, offers a robust and innovative solution to this challenge [ 40, 41]. We speculate that these approaches will soon enable the inference of physiological and pathological conditions from immune memory.

This review aims to provide an overview of the current progress in investigating antibody repertoire features and their applications, with a focus on advances in ML-assisted approaches. We also discuss future prospects and challenges in this field.

## Antibody Features that Define a Clonotype

Proteins with similar amino acid sequences tend to have similar structures and functions. After a naïve B cell is activated by an antigen in a germinal center, it undergoes massive proliferation and selection, becoming memory cells and/or plasma cells [11]. The clonally expanded cells belong to the same lineage and express antibodies with similar sequences that originate from the same unmutated common ancestor (UCA). Antibodies from the same lineage are grouped into a clonotype that can be defined by antibody sequence features.

### V gene segments

The V gene segment is the longest segment of antibody variable region and encodes two highly variable loops, CDR1 and CDR2. The International Immunogenetics Information System (IMGT) catalogues a substantial repertoire of functional human V gene segments: 56 for the heavy chain (IGHV), 41 for the kappa light chain (IGKV), and 33 for the lambda light chain (IGLV) [42]. Collectively, these gene segments contain over 400 allelic variants, contributing immensely to antibody diversity. With a growing repository of characterized antibody sequences and their cognate antigens, researchers are revealing links between V gene segment usage (V-usage) and antibody function.

#### Biased usage of V gene segments

Antibodies that recognize the same epitope within an antigen tend to use limited V gene segments, as exampled by anti-virus **neutralizing antibodies (NAbs)**. V gene segments with relatively low baseline usage enrich in those groups of antibodies, indicating a strong functional selection ( Figure 1B). For example, IGHV1-2, IGHV1-69, and IGHV4-34 are often found in anti-human immunodeficiency virus (HIV) NAbs, and IGHV1-18 is often found in anti-influenza A virus (IAV) NAbs, each accounting for over 10% of the specific antibody sequences [ 43– 45]. Structural analyses further define the functional links between V gene segments and specific epitopes, such as IGHV3-53/66 and IGHV1-58 for the severe acute respiratory syndrome coronavirus 2 (SARS-CoV-2) spike receptor-binding domain (RBD) class 1 epitope, IGHV6-1 for the IAV hemagglutinin (HA) stem, and IGHV5-51 for the third variable (V3) loop of HIV-1 gp120, which can be detected in the antibody responses of multiple individuals [ 46– 50]. Light chain V-usage demonstrates a similar selectivity, particularly in association with specific IGHV segments [ 51, 52]. Single-cell sequencing of millions of B cells reveals that compared to naïve B cells, memory B cells exhibit a greater tendency for identical heavy-light chain pairs [53]. Furthermore, since the diversity of light chains is about four orders of magnitude less than that of heavy chains [14], cognate light chains have been described in functional antibody repertoire [ 53, 54]. In addition, some V gene segments with abnormal usages have been claimed to be closely linked to autoimmune diseases [ 55, 56].

The V-usage shifts seen in functional antibodies are typically difficult to capture at the repertoire level. Thus, V-usage analysis should be combined with other immunological characteristics like antibody isotype, clonal expansion, SHM, population distribution, and longitudinal changes [57].

#### Functional contribution of germline V gene segments

The V gene segment codes ~80% of the variable region and contributes to antibody function through its unique sequence. Functional antibodies with few SHM were observed in acute viral infections and they were encoded by certain V gene segments, suggesting that germline V gene backbone can naturally recognize diverse antigens [ 58, 59] ( Figure 1B). A systematic study by Shrock and colleagues revealed germline-encoded amino acid-binding (GRAB) motifs on both heavy and light chain V genes that mediate recognition of specific epitopes through profiling a set of antibody-antigen complex structures [60].

IGHV1-69 encodes high-affinity antibodies targeting many viruses, including IVA, HIV, respiratory syncytial virus (RSV), hepatitis C virus (HCV), and SARS-CoV-2 [ 61– 66 ]. The unique hydrophobic HCDR2 of germline IGHV1-69, with Ile53 and Phe54 at the loop tip, is thought to have an innate capacity for hydrophobic contact [ 61– 63, 66]. It facilitates faster antigen recognition during acute infection than SHM-induced contact. The binding activity depends on residues in the HCDR2 loop that exert into the conserved hydrophobic pocket of the protein antigen. Of the NAbs targeting SARS-CoV-2 spike RBD, IGHV3-53 and the closely related IGHV3-66 (one amino acid difference in FR1) are the most frequently used V gene segments and their germline-encoded features, such as an NY motif in HCDR1 and an SGGS motif in HCDR2, are critical for RBD binding [ 47, 67]. Similarly, the Trp33 residue in HCDR1 and the Asp54 and Asp56 residues in HCDR2 of the germline IGHV5-51 are involved in binding to multiple antigens, including the V1/V2 domain and the V3 domain of HIV-1 gp120, and the SARS-CoV-2 RBD [ 68– 70].

Furthermore, V gene polymorphism contributes to genetic diversity in the antibody repertoire and influences individual antibody responses to infection ( Figure 1B). IGHV1-69 exhibits the greatest allelic variation, with its alleles demonstrating significant variety in antigen binding. Evidence shows that IGHV1-69 alleles with Phe at residue 54 are preferentially used in anti-IAV broadly neutralizing antibodies [71]. IGHV2-5 has 10 alleles that use either Asp or Asn at residue 54. Asp54 (IGHV2-5*02) forms stronger binding with the antigen residue by a salt bridge than Asn54 (IGHV2-5*01), as reported in anti-SARS-CoV-2 RBD and anti-HIV-1 gp41 antibodies [ 72, 73]. IGHV1-2*02 with Trp at residue 50 is necessary for encoding VRC01-class antibodies that can bind to the conserved HIV-1 gp120 epitope [74] .

### CDR3

The CDR3 regions of heavy and light chains are formed by the joining of germline gene segments, followed by deletion and/or insertion of non-template/palindromic nucleotides at the junctions [ 12, 75 , 76]. This process renders CDR3s the most diverse regions, which substantially contributes to antigen recognition. Although the comprehensive elucidation of CDR3 sequence-function relationships remains an active area of research, accumulated data from antigen-specific antibodies and antibody repertoires have revealed features that are highly correlated with specific antigen stimulation or immune states.

#### CDR3 amino acid length distribution

The length of HCDR3 is determined by various factors. On one hand, it is associated with the corresponding D and J gene segments [77]. Human IGHJ6 is longer than the other five J gene segments. Analysis of antibody repertoires in COVID-19 patients has shown an increase in the average HCDR3 length, accompanied by a significant rise in the usage of the IGHJ6 [ 78, 79]. For D gene, beyond segment length variations, they can generate long CDR3s through tandem D-D fusions that are attributed to the presence of symmetrical recombination signal sequences (RSSs) flanking the D gene [ 80, 81]. On the other hand, non-template nucleotide insertion, known as N-addition, also contributes to the length of CDR3. Public antibody clones, which are shared among different individuals, tend to have shorter CDR3s with shorter N-additions [82]. Moreover, SHM has been hypothesized to explain the generation of extremely long CDR3s, as showcased by the broadly neutralizing antibodies [83].

In the human immune system, antibodies with long CDR3s are likely to be autoreactive or polyreactive and B cells expressing such antibodies are typically eliminated during B cell maturation [84]. This selective pressure contributes to a bias in the CDR3 length distribution across the entire B cell receptor repertoire, which has a mean length of 15 amino acids for HCDR3 and 9 amino acids for LCDR3 [ 14, 85 ]. However, in patients with autoimmune diseases, such as systemic lupus erythematosus (SLE), IgG/IgA memory B cells often express antibodies with long CDR3 [55].

CDR3 length is closely correlated with antibody function, particularly with regard to the antigen epitope ( Figure 1C). Short CDR3s generally recognize flat, continuous surface epitopes, whereas extremely long CDR3s form small protrusions that bind to cryptic, recessed antigenic epitopes. Antibodies with long HCDR3s constitute a significant proportion of broadly neutralizing antibodies against HIV and play a key role in recognizing various HIV strains and surface proteins [86]. Bovine antibody genes can encode CDR3s up to 40–70 amino acids long. These CDR3s form unique antigen-recognition structures that are characterized by a stalk and a disulfide-bonded knob [87]. The extended knob of an ultralong CDR3 can bind viral antigens independently of the complete antibody structure, making it a smaller antigen-recognition unit than nanobodies (VHHs) [88] .

#### Sequence motif and preference in paratope

Many studies have highlighted the enrichment of specific amino acid motifs within the antibody CDR3 region for antigen recognition ( Figure 1C). These **paratope** motifs typically originate from germline D gene segments and are often present in antibodies that bind to the same epitope. For example, several cross-reactive antibodies that recognize a conserved site on the RBD of SARS-CoV and SARS-CoV-2 share the “YYDxxG” motif, encoded by IGHD3-22 [ 89, 90]. V gene segments of these antibodies are derived from the IGHV3, IGHV4, and IGHV1 families, and pair with diverse light chains. This makes them a class of antibodies whose binding is primarily driven by a germline D gene segment. Similarly, another class of neutralizing antibodies targeting a cryptic RBD epitope has the “WLRG” motif in the HCDR3 as paratope [ 52, 91]. Within CDR3 paratopes, amino acids such as Tyr, Gly, Asp, Ser, Arg, and Asn are more abundant than in the proteomics [92]. Aromatic amino acids (such as Tyr) can form π-π stacking with the residues on the antigen epitope. Their bulky side chains promote the structural rigidity of the CDR3 loop, which helps stabilize the antigen-binding conformation. Conversely, small-volume amino acids (such as Ser and Gly) can increase the flexibility of the CDR3 loop, allowing it to adapt to diverse epitopes [ 93, 94]. In addition, positively charged amino acid Arg can form multiple hydrogen bonds and salt bridges with negatively charged epitope residues. However, the enrichment of Arg in CDR3 can also lead to the generation of autoantibodies that bind negatively charged DNA [95]. The amino acid composition of CDR3 paratopes is also a result of antigen-driven selection. For example, aromatic and hydrophobic residues are prevalent in CDR3s of antibodies that target heavily glycosylated viral epitopes on HIV and influenza surface proteins [ 96, 97]. Together, these paratope motifs and amino acid preferences serve as molecular signatures for epitope specificity prediction.

## Clonotype Features in the Antibody Repertoire

**Figure 2:**
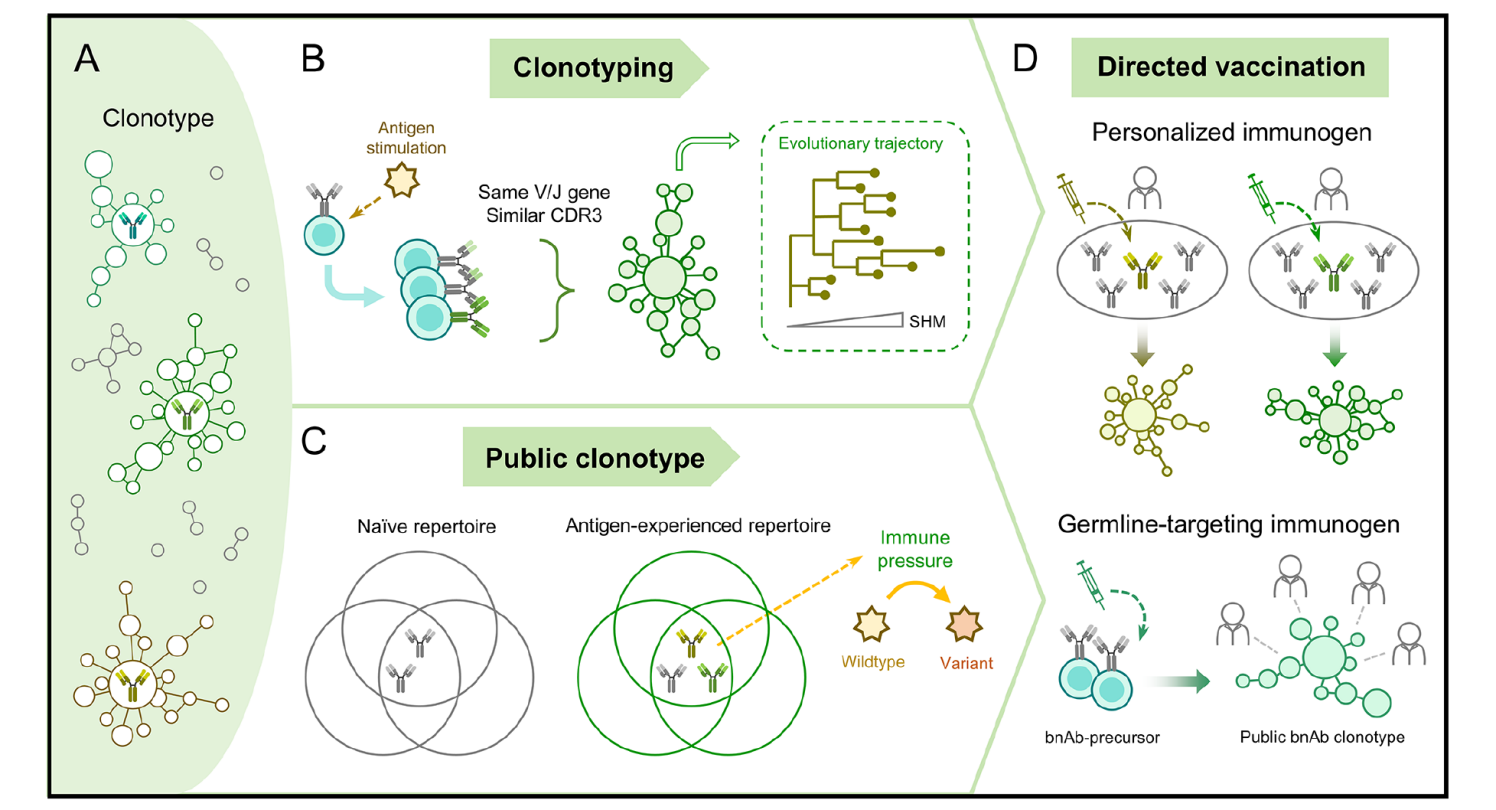
Figure 2 Clonotype features in antibody repertoires guide vaccine design strategies (A) Antibody repertoire is composed of diverse clonotypes. (B) Upon antigen encounter, naïve B cells undergo clonal expansion and affinity maturation. B cells derived from the same UCA express antibodies with related sequences and can be grouped into a clonotype based on shared V/J gene usage and similar CDR3 regions. Phylogenetic trees illustrate the evolutionary trajectories of clonotypes. (C) Public clonotypes are identified in both naïve and antigen-experienced antibody repertoires. Convergent B cell response to specific epitopes may drive viral immune escape. (D) Directed vaccines can be designed to activate desired clonal lineages within an individual (personalized immunogen) or to target shared B cell precursors capable of maturing into broadly neutralizing antibodies (bnAbs) across populations (germline-targeted immunogen).

The antibody repertoire is a vast collection of antibody clonotypes. During the immune response, antigenic stimulation shifts the distribution of clonotypes within the antibody repertoire, leading to clonal expansion and evolution of antigen-specific B cells ( Figure 2A). This process involves the B cell proliferation and SHM accumulation. The resulting cells express antibodies encoded by similar sequences, providing the foundation for clonotyping and lineage tracking, as shown in Figure 2B. Clonotype features reflect the dynamics of B cells and are closely linked to specific immune states [ 98– 100 ]. At the population level, common clonotype features across the immune response to certain diseases can be used to inform the design of novel vaccines, especially for viral infections [ 101– 104 ].

### CDR3-based clonotyping

Tracking clonal lineages within an antibody repertoire allows researchers to decipher the process of antibody affinity maturation. Currently, the criteria used to define an antibody clonotype, termed as the V3J clonotype, are V/J germline usage and CDR3 similarity [ 105, 106]. Due to the deletions and insertions in the junctions, it is difficult to distinguish CDR3 mutations generated through somatic hypermutation from those generated through V(D)J recombination in antibody repertoire sequencing data. Therefore, the CDR3 sequences are mainly used for antibody grouping and subsequent mutation analyses focus on SHMs in V gene segments [ 38, 107 ]. In V3J clonotyping, CDR3 similarity is typically determined by the edit distance between sequences, with thresholds ranging from 90% to 100% at the nucleotide-level and from 80% to 100% at the amino acid-level [108]. Both Hamming distance and Levenshtein distance have been applied in different studies. The former strictly requires identical CDR3 lengths, making it suitable for inferring clonal lineages. The latter allows for insertions and deletions and is more appropriate for mining sequences in an antibody repertoire that have functions similar to known specific antibodies. Since not all amino acids in the CDR3 directly participate in antigen binding, a more lenient similarity threshold helps to observe common functional motifs across the CDR3s of different antibodies [ 32, 91].

Within a V3J clonotype, the evolutionary trajectory of antibodies can be traced through somatic hypermutation that is strongly selected for enhancing binding to antigen ( Figure 2B). Antibody affinity maturation is understood to be a stochastic process involving mutations in the variable region exons that are selected in a Darwinian manner within germinal centers [11]. The extensive accumulation of SHMs is usually caused by sustained or sequential antigen stimulation. A classic exemplar comes from longitudinal studies of HIV-1 infected individuals, which perform long-term tracing of the neutralizing antibody clonotypes that develop extraordinary levels of SHMs for broad neutralization and co-evolve with virus [ 109, 110]. This phenomenon is also prevalent in other viral infections and vaccinations, particularly repeated immunization with heterologous antigens [ 111– 113 ]. CDRs are mutation-rich areas, augmenting binding affinity at antibody-antigen interface. Nevertheless, mutations occurring in FWRs hold comparable importance, as they can compensate for destabilizing effect from CDR mutations and increase the flexibility of the variable loops [ 114, 115].

Beyond substitution mutations, rare germline V gene insertions and deletions (indels) are present in broadly neutralizing antibodies against HIV-1 and SARS-CoV-2. Studies suggest that indel mutations are more prevalent in antibody repertoires of HIV patients than those of healthy individuals, often correlating with a high mutation rate [ 116, 117 ]. The indel distribution closely resembles that of point mutations, predominantly found in the CDR1 and CDR2 loops, with the exception of a recurrent deletion in the FR3 region [ 116, 118]. Contribution of insertion mutations to enhancing antibody affinity and neutralizing activity has been validated in some antibodies, either through direct involvement in antigen interaction or by altering the conformation of the antigen-recognition region [ 117, 119]. Furthermore, antibody repertoire tracking in SARS-CoV-2 breakthrough infections has identified similar advantageous insertions at conserved positions [120].

GC B cells undergoing clonal bursts inevitably produce bystander mutations that have little to no effect on affinity once reverted [ 48, 121]. Hence, tracing mutation pathways within antibody lineage trees is essential for pinpointing beneficial mutations. In addition, some studies emphasize the importance of high-dimensional CDR3 kernel analysis for antibody function [122]. This approach goes beyond sequence-level analysis, by incorporating factors like the physicochemical properties of amino acids, structural features, affinity prediction, and clonal lineage weight within the antibody repertoire into the evaluation criteria. Advanced computational methods are required to deal with these complicated features, such as deep learning models. Current BERT-based models, including AntiBERTa, AntiBERTy, and AbBERT, have introduced a new approach to define antibody clonotypes [ 123– 125]. From our preliminary tests (Meng *et al*., unpublished data), these models can fully capture the clonotype information but they fail to outperform traditional bioinformatic algorithms such as MiXCR [126] in this task.

### Public clonotype and convergent immune response

The enormous size of the antibody repertoire far exceeds the capacity of current measurement methods. Therefore, measured antibody repertoires vary significantly between individuals, even between monozygotic twins, with an overlap of only about 1% and relatively higher overlaps for twins [ 32, 127, 128 ]. Furthermore, sampling the same individual at different time points, from different anatomical sites, or even repeatedly sampling from the same site at the same time point, always yields a low degree of overlap among different measurements [129]. Thus, a panel of diversity indexes was applied to estimate clone diversity in an antibody repertoire [ 130– 133]. These methods reveal novel clonotype features during immune responses.

Public clonotypes are generally defined as groups of similar or identical antibodies found in multiple individuals. Within a single individual, this concept can also extend to antibodies shared across different tissues. Through high-coverage antibody repertoire analysis, public clonotypes can be detected between any two individuals [ 14, 127 ]. Among immunologically-naïve neonates and antigen-unexposed healthy individuals, this repertoire overlap is a stochastic baseline and a natural outcome of shared germline antibody gene sets through universal antibody diversification mechanisms [ 14, 127 ]. This overlap is substantially amplified in populations exposed to the same pathogens due to affinity-driven selection toward immunodominant epitopes [ 57, 134– 136] ( Figure 2C).

Convergent B cell responses have been widely reported across various diseases with well-characterized antigens [ 57, 101, 136 – 138]. Protective public clonotypes generated during these responses serve as key indicators of an effective humoral immune response, and can be used to assess individual immunity and vaccine efficacy [134]. Dominant neutralizing epitopes, particularly those that directly bind to host cell surface receptors, are more prone to elicit these public clonotypes. However, highly transmissible and mutable pathogens, especially RNA viruses such as influenza and coronaviruses, constantly replace weakened circulating strains with variants that have successfully evaded antibody recognition [ 139, 140]. In these situations, it becomes significantly more challenging to identify specific candidates by analyzing shared population characteristics within antibody repertoires because dominant public clonotypes exert selection pressure and breakthrough infections emerge by mutating the epitopes they recognize [ 139, 141]. Even though, a small number of adaptable public clonotypes maintain resistance to the variants [ 141, 142]. Notably, not all public clonotypes contain neutralizing antibodies because non-specific bystander public clonotypes can arise during immune response [143]. Determining the origin and function of public clonotypes, including bystanders, is essential for identifying functional antibodies.

### Antigen-specific clonotype and directed vaccination

Longitudinal studies of antibody repertoires have identified antigen-specific clonotypes that repeatedly appear at multiple time points in samples from the same individual across different disease cohorts [ 21, 111, 144 – 146]. These persistent antigen-specific clonotypes indicate the presence of a long-lived immune memory. Upon acute infection with novel antigens, they can persist for months while undergoing affinity maturation [ 146, 147]. At a mechanistic level, specific clonotypes can be sustained by continuous antigen stimulation, as observed in chronic infections, repeated or sequential vaccination, and tumor microenvironments [ 109, 148 – 151]. They exhibit characteristics including extensive clonal expansion, high sequence similarity, and substantial SHM accumulation [ 144, 148, 152 ]. These features suggest continuous adaptation to antigenic variation through prolonged interplay between humoral immunity and pathogen invasion. Such interplay shapes the evolutionary trajectory of antibodies within a clonotype, which can be visualized as an antibody phylogenetic tree [ 107, 109].

Antigen-driven antibody evolution has informed the B cell lineage-directed vaccination strategy [ 29, 153 , 154] ( Figure 2D). Traditional vaccine design has primarily focused on the pathogen per se ( *e.g*., attenuated, inactivated or subunit vaccines) [155]. While these approaches hold promise for inducing potent immune responses against many pathogens, they fail to develop vaccines against highly mutable viruses such as HIV. A promising new approach is to design vaccines using a germline-targeting immunogen that activates particular naïve B cells and elicits neutralizing antibodies belonging to a public clonotype which can confer protection for population. Structural analysis of epitopes that are targeted by public clonotypes can aid the design of such immunogens and enhance vaccine universality. Early trials are already underway in HIV vaccine development, as exemplified by eOD-GT8 and GT1.1 [ 103, 156]. Another approach is to map the unique antibody clonotype profiles of individuals to enable personalized vaccine strategies. These tailored vaccines could activate existing B cells with potential specificity and direct their affinity maturation to provide rapid and efficient immune protection. For older people in particular, discerning useful memory B cells from their extensive immune history is critical for designing such custom immunogens [ 157, 158 ]. Furthermore, for pathogens such as influenza viruses which present challenges due to immune imprinting, immunogens can be designed to activate naïve B cells with the potential to initiate broadly neutralizing antibody responses, or activate cross-reactive memory B cells that bind to conserved epitopes, thereby reducing the negative influence of pre-existing memory [ 159, 160].

Nonetheless, the success of these directed vaccine design strategies depends on a substantial accumulation of data, particularly regarding antigen epitope distribution and antibody paratope characteristics derived from antibody-antigen structural analyses. The number of antibody-antigen structures in databases, like Protein Data Bank (PDB) [161], remains insufficient to fully meet these demands. A summary from the SAbDab database shows fewer than 4000 non-redundant structures are currently available [162]. Therefore, the advancement of AI models will be crucial in bridging this data gap.

## Application of Machine Learning in Antibody Repertoire Analyses

**Figure 3:**
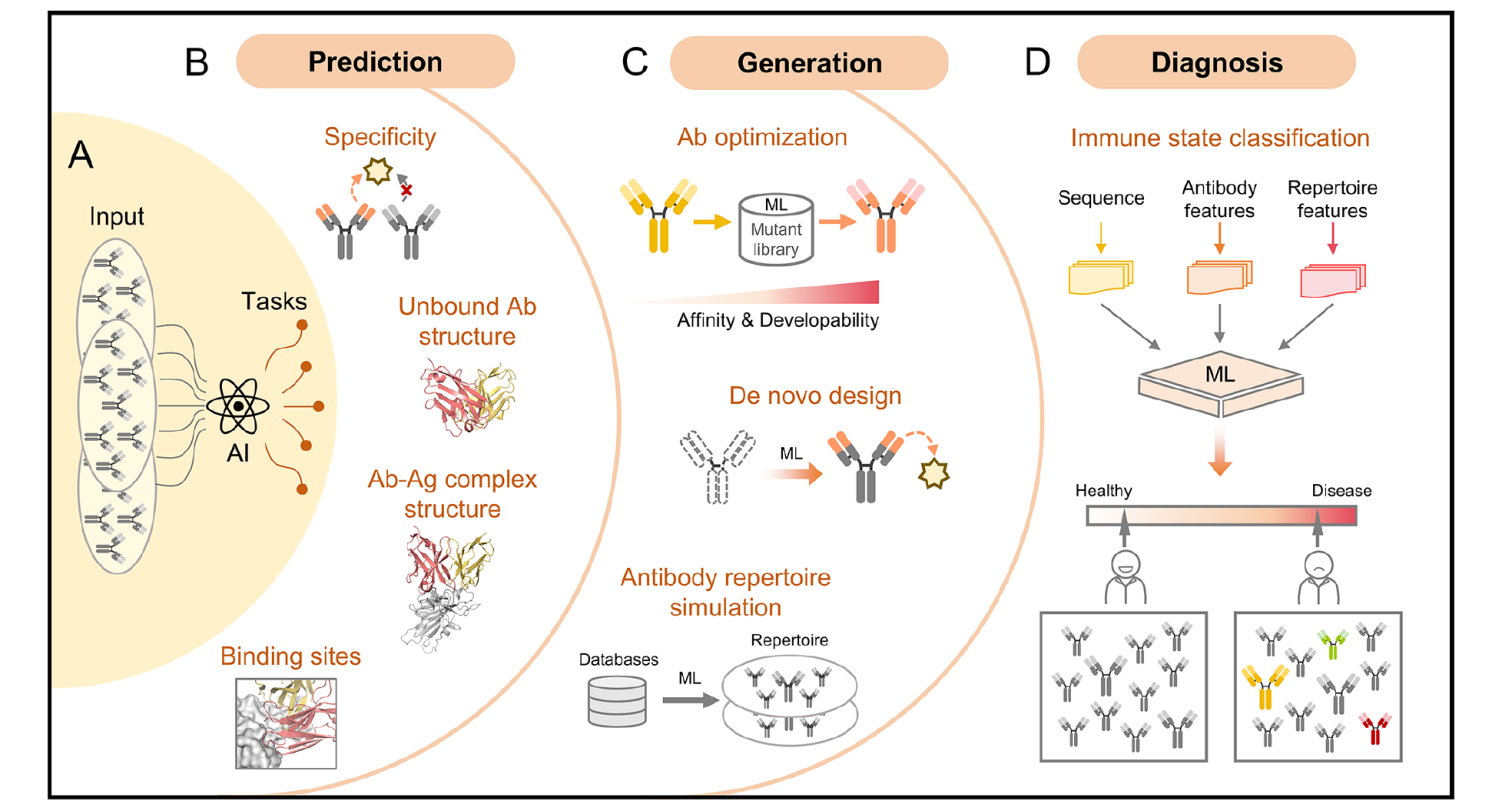
Figure 3 Application of machine learning in antibody repertoire analysis (A) Antibody sequence and structural information can be leveraged by AI models to support multiple research tasks. These include: prediction of antibody (Ab) specificity, unbound antibody structures, antibody-antigen (Ab-Ag) complex structures, and epitope-paratope binding sites (B); generation of optimized antibodies with improved affinity and developability, de novo designed antibodies for specific antigens, and simulated antibody repertoires (C); and diagnosis of immune states (D).

Antibody repertoire is shaped by the intrinsic antibody diversification and extrinsic antigen selection. Vice versa, the information revealed by the antibody repertoire features can be used for therapeutic antibody screening, biomarker discovery, and vaccine design, which collectively contribute to the prevention, diagnosis and control of diseases. However, the major challenge in recent decades has been extracting meaningful antigen information from the massive antibody sequencing data. Links between antibody repertoire and corresponding antigens have been lacking [163]. Recently, advances in deep learning (DL) algorithms have improved the interpretation of antibody repertoire features, enabling applications such as specificity prediction, antibody design, and disease diagnosis [ 10, 124 , 164– 167] ( Figure 3 A).

### Antibody-antigen structure prediction

Similar sequences or other features of antibodies may indicate convergent antigen specificity. Early efforts employed algorithms like regression, random forest, or support vector machine to predict antigen information for antibodies [ 168, 169]. Despite their limited utility in specific contexts, these methods failed to achieve this goal due to insufficient experimental structural data and restricted capacity for processing complex information.

Recently, DL-assisted antibody function prediction models have emerged ( Figure 3B). For example, DeepAAI learns functional relationships between antibodies and influenza virus antigens through adaptive graph neural networks to predict the neutralizing activity of unseen antibodies, as well as their sensitivity to new variants [170]. Similarly, mBLM trains an antibody language model with antibody-epitope data to map epitopes for antibodies with unknown targets [171]. However, a fundamental limitation of these function prediction models is the lack of structural information that precludes subtle biophysical insights and clear antigen-antibody binding interface.

High-throughput structural prediction is paramount for enhancing the accuracy and universality of antibody function prediction models. General protein structure prediction models, such as AlphaFold2 and RoseTTAFold [ 172, 173], often underperform on highly variable CDR loops, particularly CDR-H3. These CDR loops are antigen-dependent and primarily adopt binding-competent conformations in the holo (antigen-bound) state. This limits the usefulness of apo (unbound) predictions. Early prediction models relied on experimental antibody structures as templates for homology-based structural modeling and incorporated energy minimization for stability ( *e.g*., ABodyBuilder, and the original RosettaAntibody) [ 174, 175 ]. These models struggled with low-homology antibodies and failed to account for antigen-induced CDR conformational changes. In recent years, deep learning algorithms have enabled significant progress in structural prediction. As increasingly vast antibody repertoire sequencing data and structural datasets are incorporated into training, these models develop powerful extrapolation capabilities. ABlooper and DeepAb, both of which were trained on the Structural Antibody Database (SAbDab) [162], focus specifically on improving the accuracy of CDR loops, especially CDR3s [ 164, 176, 177]. AntiBERTy is a pre-trained language model trained on 558 million natural antibody sequences from the Observed Antibody Space (OAS) database [ 124, 178]. It can be fine-tuned to build models for specific tasks, such as IgFold for predicting full-length variable regions [179]. Predicting antibody-antigen binding interface routinely requires molecular docking on unbound structures. To minimize cumulative errors inherent in this two-step process, recently developed models directly learn holo-state structural information and perform sequence-to-complex prediction, such as HelixFold-Multimer and tFold-Ag which are fine-tuned from pre-trained protein-protein interaction models AlphaFold-Multimer and ESM-PPI, respectively [ 180, 181]. AbEpiTope-1.0 is further built upon the results of complex structure prediction. It enables accurate evaluation of interaction interface and precise identification of antibody binding sites on antigens, thereby achieving a higher level of function prediction [182]. Its core algorithm relies on inverse folding models, such as ESM-IF1 and ProteinMPNN, which generate amino acid sequences from given protein structures [ 183, 184].

### Antibody optimization and design

Affinity maturation was traditionally performed using time-consuming and resource-intensive directed protein evolution techniques. Deep learning algorithms have profoundly accelerated this process. Well-designed deep learning architectures streamline protein engineering by employing in silico predictions to isolate desired variants from large libraries produced by generative models, as shown in Figure 3C. Commonly used generative models include: (1) variational autoencoders (VAEs), which learn the latent space of protein data and then use it to generate diverse new proteins [185]; (2) generative adversarial networks (GANs), which use a generator and a discriminator to produce highly convincing protein candidates or simulate protein repertoires [186]; (3) diffusion models, which generate high-quality data from random noise through iterative denoising and are well-suited for structure generation [187]; and (4) protein language models (PLMs), which learn the grammar of biological language from massive protein sequences to predict missing or next amino acids in a sequence [188].

Many studies have already developed DL-based antibody optimization models that yield engineered variants with markedly improved binding affinity relative to parental sequences [ 166, 189 – 191]. Emerging antibody language models ( *e.g*., IgBert) further refine this process by explicitly modeling heavy-light chain cooperativity. This facilitates the design of antibodies with optimized biophysical properties, enhanced structural stability and functional coordination [192]. However, deep learning models are fundamentally restricted by the availability of high-quality labeled training data. The Design-Build-Test-Learn (DBTL) framework overcomes this limitation by using iterative optimization to progressively expand the training dataset with experimentally validated sequences from initially unlabeled in silico-generated pools [193]. This process has the potential to improve the sequence-to-function prediction. In addition, inverse folding models enable efficient antibody optimization by leveraging structural information to predict sequences that maintain complex stability and enhance function, achieving significant improvements without requiring task-specific training data [194].

Notably, antibody optimization encompasses not only binding affinity and specificity, but also the comprehensive improvement of developability attributes, including expression yield, immunogenicity risk, thermal stability, and solubility, which are critical for clinical translation [ 195– 197]. Due to its capacity for high-dimensional pattern recognition, deep learning is uniquely suited for this multi-parameter optimization challenge. The key to achieve balance among the competing objectives is to accurately predict multi-dimensional performance and quantify uncertainty, and then use intelligent multi-objective optimization algorithms ( *e.g*., Bayesian optimization) and iterative learning to pinpoint the best solution on the Pareto front [198].

Another revolutionary application of deep learning is *de novo* antibody design, which is a special form of binder protein design, for example RFantibody and Chai-2 [ 199, 200 ]. Both approaches successfully design antigen-specific VHHs or single-chain variable fragments (scFvs), but differ in methodology and focus. RFantibody employs a binder-first paradigm, generating VHHs to fit pre-defined epitopes with high structural precision and an ~80% validation rate. However, the requirement for large structural datasets and fine-tuning makes it computationally intensive and less scalable for new targets [199]. In contrast, Chai-2 employs an epitope-first paradigm, generating all CDRs from scratch based solely on target epitope information. This zero-shot approach requires minimal data and achieves a rapid design cycle (under two weeks) with a notable 16% antibody hit rate [200]. While less structurally precise, it is more efficient and is applicable for targets lacking known antibodies. More comparison information of these two approaches is shown in Table 1.

**Table:** **Table 1** Comparison of *de novo* design models

Comparison	RFantibody	Chai-2
Design paradigm	Binder-first	Epitope-first
Core models	Fine-tuned RFdiffusion (backbone)ProteinMPNN (sequence); Fine-tuned RoseTTAFold2 (validation)	Multimodal generative model
Inputs	Target antigen structure and a specified epitope (a pre-existing antibody framework is needed)	Target antigen structure and a specified epitope
Success criteria	Nanomolar affinity, structural accuracy	16% antibody hit rate, picomolar-nanomolar affinity
Wet-lab validation	~80% hit rate (4/5 VHHs)	16% hit rate of ≤ 20 binders for each target, 50% of 52 targets with ≥ 1 binder
Advantages	High structural precision	High efficiency and scalability
Disadvantages	Requirement for extensive structural datasets and computing resources	Lower structural precision

At the level of antibody repertoire, classical probabilistic models, such as IGoR and immuneSIM, are commonly used to simulate repertoires by modeling V(D)J recombination as sequential events (gene selection, nucleotide insertion/deletion) [ 201– 203 ]. These models produce biologically realistic sequences with interpretable generation probabilities, but are short of solving complex non-linear patterns ( *e.g*., paired heavy/light chain correlations, disease-specific repertoire signatures). While DL-based generative models are not yet widely applied in repertoire simulation, they could potentially capture intricate high-order correlation within sequences, enabling the generation of novel, biologically plausible sequences and providing mechanistic insights into antibody diversification ( Figure 3C). Again, high-quality paired heavy-light chain antibody sequences and paired antibody-antigen data are required for model training.

### Disease diagnosis using antibody repertoire sequence data

Antibody repertoire sequencing provides a solid foundation for decoding humoral immunity. In addition to therapeutic antibody discovery, these datasets capture the history of immune system, revealing both transient fluctuations and chronic adaptations associated with disease or aging ( Figure 3D). Early studies have attempted to distinguish immune states using statistical features of antibody repertoires, with the aim of obtaining biomarkers for diverse detection scenarios [25]. Commonly used statistical features fall into two categories: sequence-level features and clonotype-level features. The former includes V(D)J gene usage, CDR3 length and amino acid composition, and SHM frequency [ 204, 205], which correlate with antibody function. The latter primarily involves clonotype diversity, persistence, and sharing [ 205, 206], which represent a systematic evaluation of B cell dynamics. Evidently, classification models that exclusively rely on macroscopic statistical measures fail to capture high-dimensional information coded in the antibody repertoire, resulting in their accuracy and generalization capabilities being unable to meet current application requirements.

DeepID, a DL-based diagnostic model, employs 10 repertoire-level and 160 sequence-level features to distinguish infected individuals from healthy controls via ensemble learning [207]. This approach demonstrates improved predictive accuracy while identifying signatures associated to immune states. However, DeepID suffers from the same drawback as traditional statistical feature models in that it highlights the influence of high-frequency antibodies and potentially overlooks the contributions of low-frequency clones. The immune repertoire classifier DeepRC uses a Multiple Instance Learning (MIL) framework with attention mechanisms to assign higher weights to antibody sequences that are critical for diagnosis, regardless of their abundance in the repertoire [208]. Meanwhile, Mal-ID leverages the ESM-2 protein language model to generate high-dimensional, context-sensitive embeddings for each immune receptor sequence, including B cell receptors (BCRs) and T cell receptors [10]. It combines two sub-models based on repertoire features to enhance learning efficiency [10]. This architecture enables the simultaneous diagnosis of multiple diseases by tracing the immunological fingerprints of past infections and disease histories. However, these models are still in the preliminary stage and cannot be used directly in a clinical setting. With more high-quality training data and new deep learning frameworks, future models have the potential to transform current diagnostic workflows and enable personalized therapeutics.

### High-quality antibody reactome data for model training and diagnosis

The vast diversity of the human antibody repertoire makes clonotype-level antibody sequence changes insufficient for discovering disease-specific biomarkers. A large portion of the repertoire consists of naïve B cells that are not directly engaged in the ongoing or memorized immune responses. This obscures the detection of meaningful disease-associated signals within the background. High-quality, high-throughput linked antigen-antibody data ( *i.e* ., **antibody reactome**) are required for model training.

The antibody reactome provides a functional snapshot of the immune system by identifying the specific antigens that shape immune responses in a given pathological or physiological context [209]. High-throughput profiling methods are indispensable for this task, as they enable the screening of millions of antibody-antigen interactions simultaneously. One such method is phage immunoprecipitation sequencing (PhIP-Seq), which employs bacteriophage libraries displaying diverse peptides to capture linear epitopes for antibodies from patients with autoimmune or infectious diseases [ 23, 210 – 212]. Using the PhIP-seq platform, VirScan revealed that each person carries a unique viral exposure signature and that, on average, individuals are exposed to ten viral species [213]. The same technique was also used in mapping the antibody-binding epitope upon viral infection and identifying potential biomarkers [ 212, 214]. Randomly encoded antigen profiling (REAP) utilizes yeast-displayed libraries to comprehensively map autoantibody specificity [24]. Due to its ability to present full-length, native-like extracellular proteins, REAP can detect antibodies that bind to conformational epitopes, providing more information about auto-antibody in the context of viral infection, autoimmune disease, or tumorigenesis [ 24, 215 , 216].

Reactome data have a crucial advantage over antibody repertoire sequencing data alone in that they provide direct evidence of antigen specificity. While repertoire data can demonstrate clonal expansion, it cannot identify the antigens driving that expansion. By contrast, reactome data directly link antibody clonotypes to their cognate antigens, illuminating the molecular drivers of immune responses. This functional insight facilitates targeted biomarker discovery by distinguishing disease-driving antigens from irrelevant background activity. Furthermore, once an antigen-specific antibody is identified, its sequence can be traced back within repertoire datasets, thereby highlighting otherwise obscured clonal expansions and revealing lineage dynamics. Large-scale antibody-antigen interaction datasets from the antibody reactome also serve as foundational resources for training AI models, enabling advances in prediction, design, and diagnosis.

## Perspectives

In this review, we briefly discuss the definition of an antibody clonotype and its features revealed by traditional bioinformatic approaches. We also highlight that the antibody repertoire contains crucial immune information that AI models can use to design antibodies, guide vaccine development, evaluate immune efficacy, diagnose immune states, predict disease progression, and uncover pathogen immune evasion history. AI is reshaping the current research methodology in the life science. In the face of this challenge, experimental biologists must answer critical questions such as how to collaborate to develop AI models, how to select suitable models, and which questions to address.

Currently, developing AI models requires ample computing resources and high-quality training data. The restriction on computing resources could be solved by optimizing the computing framework. The DeepSeek model demonstrates this solution. It uses an efficient mixture-of-experts (MoE) architecture and only activates the necessary parts of the model for each task during training and inference [ 217, 218]. The latter possesses a question that experimental biologists can contribute directly to. Several protein language models [ 124, 165, 219, 220] developed thus far have been trained using the same public antibody sequence data from the OAS database [178], and are therefore limited by the training data. For example, most antibody sequence data only contain heavy chain information and paired heavy-light chain sequence data are insufficient. Similarly, paired antibody-antigen information is scarce, with most data extracted from a limited set of complex structures as abovementioned. However, current antibody characterization methods cannot generate the large datasets required for model training. Therefore, there is a great opportunity for biologists to develop high-throughput, low-cost methods to obtain such data for specialized models. Heavy-light chain paired antibody sequences from single-cell sequencing and paired antibody-antigen data from antibody reactome are a good place to start antibody research.

In recent years, we have witnessed the booming of AI models in the antibody field. Each model often claims to have better predictive abilities than previous models. Thus, there is an urgent need to develop standardized ecosystems, as proposed by immuneML [221], to provide users with a unified framework for comparing, selecting, or developing AI models tailored to their specific needs. Ideally, we would ask different models to perform the same task and choose a consensus expert opinion. Nowadays, protein language models are used more frequently due to the enormous amount of antibody repertoire sequencing data. We speculate that, in the near future, more types of data will soon be applied to train antibody models, including paired antibody-antigen/paratope-epitope information, affinity, B cell transcriptomes, and immune cell interaction data. Multi-dimensional models will improve our understanding of humoral immunity.

Currently, DL-based antibody design primarily focuses on antibody optimization and *de novo* design of specific antibodies targeting known epitopes remains challenging. Multi-dimensional models that consider germline gene segment usage directed affinity maturation, and developability optimization could potentially inspire *de novo* antibody design pipelines. Another task for AI models is immune state prediction. While several diagnostic models claim to predict specific diseases with high accuracy [ 10, 207, 208 ], their performance has not yet been standardized due to the heterogeneous training data from different diseases. Looking forward, immune state prediction models should strive to recognize more disease types. These models could also be used to design customized vaccination or therapeutic strategies. We anticipate a future with more AI tools in the medical field.

## Glossary

**Table:** 

Term	Definition
Antibody reactome	The comprehensive set of specific binding events that can occur between all antibodies and their corresponding antigens within a given biological system. It reflects the unique immunological fingerprint of an individual.
Antibody repertoire	The full diversity of antibodies (or BCRs) in an individual, generated by V(D)J recombination and refined through SHM and antigen-dependent selection.
Class switching recombination (CSR)	An activation-induced cytidine deaminase (AID)-mediated deletional DNA recombination process in activated B cells that replaces the immunoglobulin heavy-chain constant region, thereby altering antibody effector functions while maintaining antigen specificity.
Germinal center (GC)	A transient structure in secondary lymphoid organs where antigen-activated B cells undergo SHM-driven diversification and affinity-based selection through interactions with follicular dendritic cells (FDCs) and T follicular helper (Tfh) cells. This process generates class-switched, high-affinity plasma cells and memory B cells, forming the basis of adaptive humoral immunity.
Humoral immunity	A B cell-driven adaptive immune response initiated by BCR recognition of native antigens. In germinal centers, SHM diversifies antibodies and affinity selection favors high-affinity BCRs. CSR alters antibody classes to tailor effector function mediated by crystallizable fragment (Fc). Plasma cell-secreted antibodies that execute immune defense via neutralization, Fcγ receptor-dependent opsonophagocytosis, and complement activation.
Neutralizing antibody	An antibody that binds to a specific pathogen and prevent it from infecting host cells and causing disease.
Paratope	The specific region within the variable region of an antibody, composed of a unique set of amino acids, that recognizes and binds to a corresponding epitope on an antigen. This interaction enables the antibody to target and neutralize the antigen with high specificity.
Somatic hypermutation (SHM)	An AID-dependent process in GC B cells that introduce high-frequency mutations into immunoglobulin variable-region genes, focusing on CDRs to enable affinity maturation through selection.
V(D)J recombination	A DNA recombination process mediated by the recombination-activating gene (RAG)1/2 complex, which assembles V, D, and J gene segments to generate diverse immunoglobulin variable regions.
